# Maternity and Mothering

**Published:** 1912-01-13

**Authors:** 


### MATERNITY AND MOTHERING.

Inquiries and Correspondence are invited.

### Nervous Vomiting in Childhood.

There are many good and sufficient reasons why children more frequently suffer from attacks of vomiting than do adults. Most children will, if they are allowed the chance, over-eat themselves on any food to which they are strongly attracted; and children, as everyone knows, are fonder than their seniors are of sweet and over-rich or "sickly" dishes. Vomiting, moreover, is a much easier and simpler proceeding in the child than in the adult; nausea and distress are often at a minimum, and emesis itself is rapid and painless.

### Peculiarities in Children.

There are, moreover, in addition, certain peculiarities of this function in children which are not at all easy to explain. Thus the initial act of vomiting at the commencement of an infectious fever, or of any acute indisposition, must be presumed to be of central, not local, origin; and so also must that peculiar disorder known as the "cyclical" vomiting of childhood. That is to say, probably that these forms of vomiting are produced by the effect of toxins or other abnormal substances upon the nervous centres. The connection of cyclical vomiting with acetonuria, and with the acidosis which may follow anæsthesia in children, is also as yet somewhat indefinite, though enough work has been done at the pathology of these elusive conditions to show that they are highly complex, and that the problems which they present are very intricate.

There is, however, another kind of vomiting which afflicts children, and must be supposed to be of central origin; for it is not caused by those local stomachic, intestinal, or appendicular dyspepsias and indigestions which are so frequent amongst juvenile patients. This, for want of any more precise definition of it, is often known as the nervous vomiting of children; and as it often causes much anxiety to parents and guardians it is important that those who encounter it professionally should be able to recognise it promptly and treat it satisfactorily. Attention has recently been directed to this not very uncommon childish ailment by Dr. E. Bellingham Smith, who gives brief accounts of seven typical cases which have been under his observation during the last two years.* This author points out the resemblances between nervous vomiting of this sort and another nervous trouble which causes endless worry to those who have charge of children, enuresis, an account of which was given in these pages recently (The Hospital, December 16, p. 283).

The salient characters of this vomiting are that it is persistent, not periodic; and that it may go on regularly for weeks or months. It most commonly takes place just after a meal, or during a meal, and happens once a day or oftener. It is painless, and there is no retching; nor are there any signs or symptoms to be found of any local condition that can account for it; nor is any evidence to be found of peripheral irritation which might excite vomiting as part of a nervous reflex. Yet another curious fact is that general nutrition suffers very little if at all; the patients do not lose weight, and do not express any sense of being ill.

There is one point in the ætiology which is significant. The children who display this type of vomiting are nearly always of nervous inheritance, and they very often themselves display other signs of nervous instability: migraine, habit spasms, nocturnal incontinence were all noticed among some or other of the children with whom Dr. Bellingham Smith's article deals. Further associations ætiologically are rheumatism and rickets. As for the ancestry, the author remarks that the mother is often of a neurotic temperament.

It need hardly be said that such cases demand the most thorough physical examination to exclude all possibility of abdominal or other disease. Many children suffer from periodical "bilious attacks," which are as a rule due to constipation or to over-eating, or to both. Such attacks are often attended by extremely little pain, but they do not recur so frequently or so regularly as do the symptoms of nervous vomiting. An unsuspected mild chronic appendicitis, too, may set up occasional attacks of vomiting in which pain is very little complained of; but there is nearly always tenderness in the right iliac fossa on careful palpation. Such cases are unhappily far from rare, and now and then they terminate in a fulminating appendicitis about which there can be, or should be, no mistake.

### Treatment.

Children who suffer from this distressing condition—distressing, that is, to their relatives, for it seems to disturb the patients themselves uncommonly little—need treatment directed mainly towards the condition of nervous instability of which vomiting is merely a sign. The injudicious coddling and solicitude of a neurotic mother is to be done away with, if necessary, by withdrawing the child temporarily from her society. A regular, healthy life, with plenty of suitable and supervised exercise, plenty of fresh air, and enough, but no more, of plain nourishing food, are important items in the treatment. The treatment of constipation is another detail which demands attention. Then as for drugs, a simple form of tonic treatment, adapted to the circumstances of the particular case, will answer best. Dr. Bellingham Smith recommends small doses of arsenic and opium given just before meals; and atropine treatment should enuresis be a factor in the case. It is also obvious that if the patient is rickety he should be treated with cod-liver oil, phosphates, hypophosphites, or lime salts, according to his requirements. If he be anæmic, some ferruginous tonic will be indicated.

Simple treatment along these lines will nearly always prove speedily beneficial.

* *Lancet*, December 23, 1911, p. 1769.

